# Ectoine Globally Hypomethylates DNA in Skin Cells and Suppresses Cancer Proliferation

**DOI:** 10.3390/md21120621

**Published:** 2023-11-29

**Authors:** Majjid A. Qaria, Chunyan Xu, Ran Hu, Roua A. Alsubki, Mohamed Yassin Ali, Sethupathy Sivasamy, Kotb A. Attia, Daochen Zhu

**Affiliations:** 1 Biofuels Institute, School of the Environment and Safety Engineering, Jiangsu University, Zhenjiang 212013, China; majidqaria@gmail.com (M.A.Q.); xuchunyanjsu@sina.com (C.X.); mya02@fayoum.edu.eg (M.Y.A.); sethulifescience@gmail.com (S.S.); 2 School of Medicine, Jiangsu University, Zhenjiang 212013, China; hr_220322@163.com; 3 Department of Clinical Laboratory Science, College of Applied Medical Sciences, King Saud University, 2455, Riyadh 11451, Saudi Arabia; ralsubki@ksu.edu.sa; 4 Department of Biochemistry, Faculty of Agriculture, Fayoum University, Fayoum 63514, Egypt; 5 Department of Biochemistry, College of Science, King Saud University, 2455, Riyadh 11451, Saudi Arabia

**Keywords:** ectoine, epigenetic modifications, DNA hypomethylation, skin cells, tumorigenicity, cell proliferation, DNMTs, anti-aging, cancer

## Abstract

Epigenetic modifications, mainly aberrant DNA methylation, have been shown to silence the expression of genes involved in epigenetic diseases, including cancer suppression genes. Almost all conventional cancer therapeutic agents, such as the DNA hypomethylation drug 5-aza-2-deoxycytidine, have insurmountable side effects. To investigate the role of the well-known DNA protectant (ectoine) in skin cell DNA methylation and cancer cell proliferation, comprehensive methylome sequence analysis, 5-methyl cytosine (5mC) analysis, proliferation and tumorigenicity assays, and DNA epigenetic modifications-related gene analysis were performed. The results showed that extended ectoine treatment globally hypomethylated DNA in skin cells, especially in the CpG island (CGIs) element, and 5mC percentage was significantly reduced. Moreover, ectoine mildly inhibited skin cell proliferation and did not induce tumorigenicity in HaCaT cells injected into athymic nude mice. HaCaT cells treated with ectoine for 24 weeks modulated the mRNA expression levels of *Dnmt1*, *Dnmt3a*, *Dnmt3b*, *Dnmt3l*, *Hdac1*, *Hdac2*, *Kdm3a*, *Mettl3*, *Mettl14*, *Snrpn*, and *Mest*. Overall, ectoine mildly demethylates DNA in skin cells, modulates the expression of epigenetic modification-related genes, and reduces cell proliferation. This evidence suggests that ectoine is a potential anti-aging agent that prevents DNA hypermethylation and subsequently activates cancer-suppressing genes.

## 1. Introduction

Epigenetic modifications are mainly driven by DNA methylation, and DNA hypermethylation is strongly associated with cancer development and aging [1]. Drugs like azacitidine and decitabine derivatives of 5-aza-deoxycytidine (5-aza-CdR) selectively inhibit DNA methyltransferase1 (DNMT1), hypomethylate DNA, and reprogram tumor cells to a normal-like state [2]. However, these drugs have serious side effects such as severe skin toxicity [3]. Several drugs targeting epigenetic modifications have been developed to target cancer that showed several drawbacks that prevent their application owing to serious adverse reactions, such as HDACi [1,4]. Historically, natural compounds have been of interest for the treatment of idiopathic diseases such as allergies and cancers because of their safety record. Among these, naturally produced compatible solutes/osmolytes, including some amino acids and their derivatives, have been proven to be critical in protecting organisms from increased environmental abiotic and biotic stresses [5]. In 1985, Galinski et al. characterized the well-established flagship and novel amino acid derivative ectoine (1,4,5,6-tetrahydro-2-methyl-4-pyrimidine carboxylic acid) osmolyte as a new component present in marine microorganisms such as the extremely halophilic bacterial genus *Ectothiorhodospira* [6,7,8,9,10,11]. Ectoine is considered to be an excellent DNA protectant against environmental biotic and abiotic stresses, ultraviolet (UV) radiation, and other types of radiation. Ectoine protects the protein’s water shell due to preferential exclusion from the immediate hydration shell of the proteins caused by the osmophobic effect [12]. Another interesting ectoine protection mechanism is through scavenging hydroxyl radicals and getting converted into N-acetamide aspartate and N-acetamide-β-alanine [13]. Owing to the fascinating features of ectoine (low molecular weight, zwitterionic, non-cytotoxic, strong water binding, and cyclic amino acids), it has been used in numerous healthcare applications in the healthcare and medical sectors, e.g., as therapeutic agents for allergies, inflammation, and cancer alleviation [5,14,15,16,17,18,19]. Ectoine has poor bioavailability in eukaryotic cells, and treatment of RAW cells with ectoine showed that 97% of ectoine was in a medium and only 3% was in cell fraction [20]. However, several studies have studied the in vitro activities of ectoine in eukaryotic cells without considering the hydrophilic character of ectoine [21,22,23].

Epigenetic events such as aberrant DNA methylation have been shown to silence the expression of many genes that suppress malignancy, including skin cancer. DNA cytosine methylation is one of the best-studied epigenetic modifications and has been proposed to play a key role in multiple biological processes and the pathogenesis of disease conditions [24]. The primary mechanism of DNA methylation involves the transfer of methyl groups from S-adenosylmethionine (SAM) metabolites through covalent modification, with one carbon atom attached to the fifth position of cytosine to form 5-methyl cytosine (5mC) [25]. This covalent modification is mainly driven by the DNMTs such as DNMT1, DNMT3a, and DNMT3b. DNMT1 maintains the methylation reaction during the replication phase, and the other two DNMTs perform de novo methylation [26]. DNMT3-like (DNMT3L) is a protein that regulates Dnmt3a, and its co-expression significantly enhances the de novo methylation by Dnmt3a. Dnmt3a stimulation is noticed at maternally methylated imprinting centers (ICs) such as small nuclear ribonucleoprotein polypeptide N (SNRPN) [27]. There is another major epigenetic modification called histone deacetylation that is driven by histone deacetylases (HDACs), and the aberrant expression of these genes is related to various types of cancer development, including melanoma [28,29]. Another emerging critical methylation mechanism is the methylation of N6-adenosine (m6A) by methyltransferases, mainly methyltransferase-like 3 and 4 (METTL3, METTL4), which adds m6A to target RNAs [30]. Hypermethylation of one of the imprinted genes, mesoderm-specific transcript homolog (MEST), was observed in males with oligozoospermia and idiopathic male infertility [31]. Further, lysine-specific histone demethylase 3 (KDM3) belongs to the histone demethylases family and has been shown to induce gene expression [32].

It is known that ectoine is widely used in skincare products and allergies and inflammation therapies. Radiations cause DNA hypermethylation [33], and on the other hand, ectoine protects DNA well against ionizing radiations [34,35]. An in vitro study showed that ectoine protected the mitochondrial DNA from UVA [22], while other studies showed that ectoine enhanced the single strand breaks and changed the conformation of coiled DNA [36,37]. In this context, understanding the molecular mechanisms behind ectoine’s therapeutic potential against several diseases and the study of the epigenetic modification prospects need to be further investigated. Determining the molecular mechanisms underlying the effectiveness of ectoine as a DNA protectant is necessary to highlight the roles of compatible solutes in protecting cells from environmental stress that causes DNA instability and to better understand senescence and cancer prevention.

In this study, we investigated the roles of ectoine in global DNA methylation in skin cells, 5-mC percentage in skin cells, and regulation of epigenetic modification-related genes and markers. We also investigated the effects of ectoine on the viability, proliferation, and functional pathways of skin cancer cells.

## 2. Results

### 2.1. Global Methylation Sequencing Revealed That Ectoine Hypomethylates DNA and Enriches Nucleotide Binding Ions DEGs

**Figure 1:**
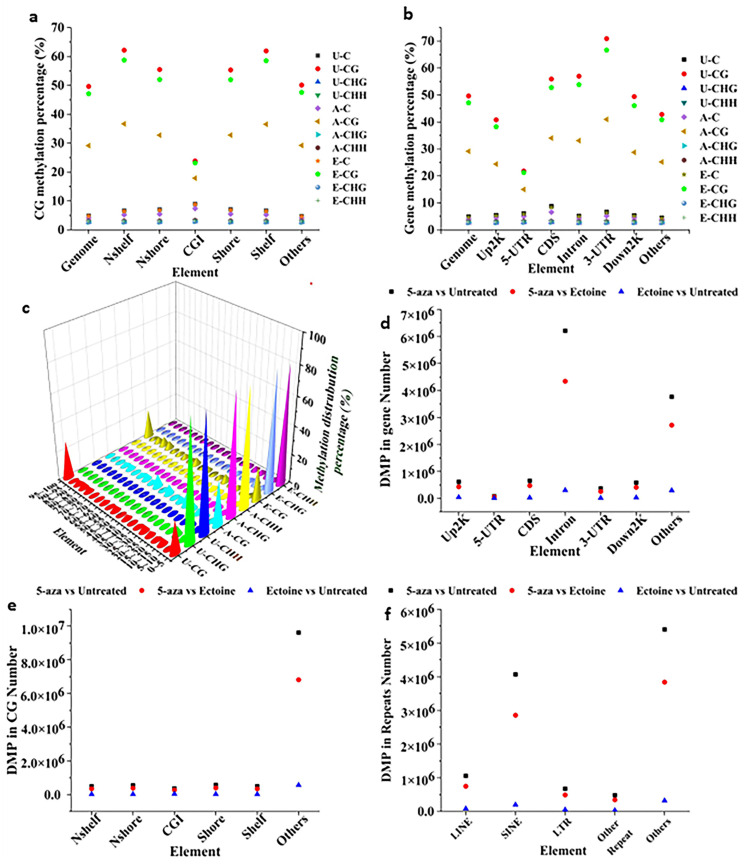
Ectoine hypomethylated DNA in skin cells. Global DNA methylation analysis in HaCaT cells treated with ectoine compared to that in cells treated with 5-aza and untreated cells subjected to global methylation sequencing. Gene methylation analysis was performed using the methylated C region and was determined from the comparison results of BisMark using a binomial distribution test. DMPs and DMR were analyzed using Bioconductor package DSS. The data show the methylation level of DNA at the CGI level (**a**), gene element methylation level (**b**), and total methylation distribution level (**c**). The DMPs’ analysis showed that the number of HaCaT DMPs in the CG elements (**d**) was similar to the number of DMPs (**e**) and repeats (**f**).

To investigate the role of ectoine interaction with DNA, we performed whole-genome bisulfate sequencing on HaCaT cells treated with either ectoine for the long term or 5-aza. Methylome analysis of ectoine-treated cells showed that CG methylation percentage, in general, was high at Nshelf and shelf and low at CGI. Ectoine-treated cells’ methylation CG percentage levels significantly reduced in almost all elements compared to untreated cells (Figure 1a). At the gene methylation level, in general, the methylation level was high at 3-UTR and low at the 5-UTR gene element. Ectoine-treated cells showed reduced methylation percentage in all gene elements compared with untreated cells (Figure 1b). Overall, genome methylation distribution in corresponding sequence contexts CG, CHH, and CHG was reduced in longer sequences (90–100) compared to untreated cells. However, in cells treated with DNMT1 inhibitor (5-aza), methylation of CG elements, gene elements, and genome methylation distribution percentage were greatly reduced compared to untreated and ectoine-treated cells. For further analysis, the differentially methylated positions (DMPs) between the groups were analyzed using Bioconductor package DSS, and it was found that the total number of DMPs between ectoine and untreated cells was 689,850, while the number of DMPs of 5-aza was 11,687,481 compared to untreated cells and between 8,277,681 compared to ectoine-treated cells. The DMPs among CG elements were the highest compared to Nshelf, Nshore, Sshore, Sshelf, and CGI elements (Figure 1d). Gene DMPs were mostly distributed in introns (Figure 1e), while in genome repeats, the SINE and others contained the highest DMPs (Figure 1f). However, in the total genome DMPs ectoine-treated cells showed significant difference irrespective of hypermethylated or hypomethylated positions. DMPs in 5-aza were very high compared to ectoine-treated and untreated regions, and the other elements were the highest compared to untreated cells. Overall, ectoine hypomethylated DNA in skin cells in almost all genome elements.

Furthermore, gene ontology (GO) enrichment functional analysis between functional pathways showed that ectoine-treated cells had lower differentially expressed genes (DEGs) of (includes biological processes (BP), molecular functions (MF), and cellular components (CC)) pathways compared to 5-aza vs. untreated cells and 5-aza vs. ectoine DEGs. Interestingly, tree-map GO enrichment analysis showed that ectoine vs. untreated ion binding, small molecule binding, nucleotide binding, and ribonucleotide binding-related DEGs were enriched compared with 5-aza vs. untreated and 5-aza vs. ectoine. Moreover, ectoine improves cellular biological processes such as cell morphogenesis. KEGG functional analysis showed that ectoine reduced the overall expression of genes compared to 5-aza vs. untreated and 5-aza vs. ectoine. Moreover, ectoine-induced morphine addiction-related genes in the cells treated with ectoine (Supplementary Figure S1a–i).

### 2.2. 5mC Methylation

**Figure 2:**
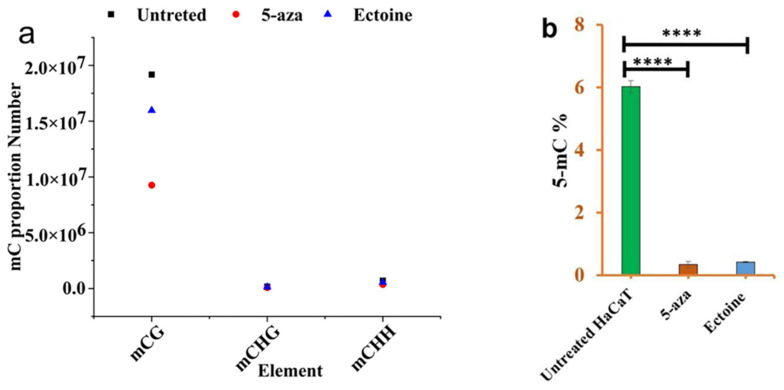
Ectoine reduced 5-mC methylation level in skin cells. Global methylation sequencing analysis showed that the 5mC proportion number in ectoine-treated cells, 5-aza-treated cells, and untreated HaCaT cells (**a**). Similarly, DNA isolated from HaCaT cells treated with ectoine or 5-aza or untreated were analyzed in vitro using a 5-mC antibodies quantification kit as per the manufacturer’s instructions (**b**). (**** *p* < 0.0001).

To validate global DNA methylation, in vitro, we analyzed the percentage of methylation-targeted 5mC in the DNA using 5mC specific antibodies and in silico analysis for the mCG, mCHH and mCHG sequence contexts proportion from the whole-genome bisulfate sequences. Bioinformatic analysis of the methylome showed that the proportion of mC was significantly lower in ectoine-treated cells than in untreated cells, while it was the lowest in 5-aza-treated cells (Figure 2a). Antibodies targeting 5mC showed that ectoine significantly hypomethylated DNA compared to untreated cells (Figure 2b). However, 5-aza-treated cells showed marked hypomethylation. Therefore, ectoine reduced the DNA 5mC level throughout the whole DNA in skin cells.

### 2.3. Ectoine Is a Non-Tumorigenic Molecule and Reduces Cell Proliferation

**Figure 3:**
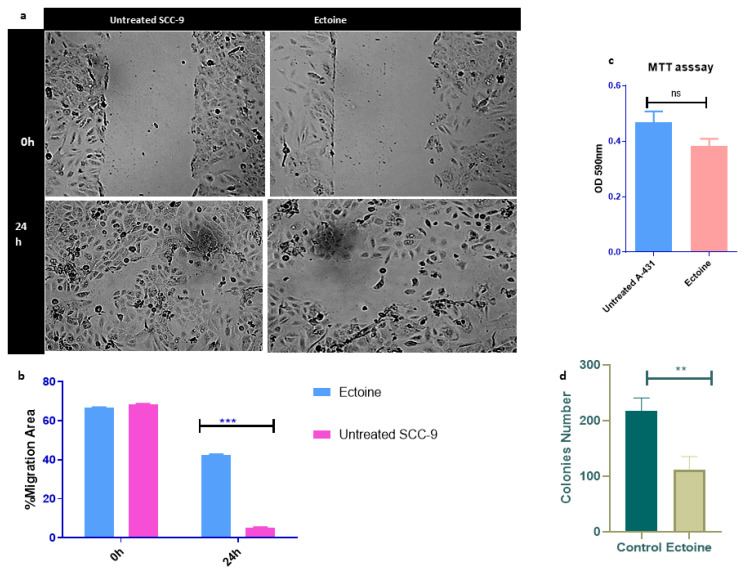
Effects of ectoine on skin cell proliferation. Wound healing assay was employed to determine the migration of SSC cells treated with ectoine and control visualized under bright field microscopy after 0 h and 24 h (*** *p* < 0.001) (**a**), and the migration areas were analyzed using ImageJ software, presented and statistically analyzed using GraphPad prism 8.0.2 and *t*-test (**b**). MTT assay to SCC cells treated with ectoine and untreated control for 48 h and visualized by plate reader after 48 h (**c**). (ns denotes *p* > 0.05). Clonogenic assay for HaCaT cells. The cells were seeded at 100 cells/mL and treated with ectoine, and formed colonies were counted after 9 days, presented, and statistically analyzed using GraphPad prism and *t*-test (** *p* < 0.01) (**d**).

To study the effects of ectoine on skin cancer cell proliferation, we performed wound healing assay, clonogenic assay, and MTT assay. Wound healing assay results showed that ectoine inhibited cell migration compared to untreated cells. The wound healed in cells untreated with ectoine after 24 h, and the cells treated with ectoine took longer to heal (Figure 3a,b). Similarly, we confirmed these results using A-431cells, and the results showed that wound healing and cell migration to close the wound were slower in ectoine-treated cells than in untreated cells (Supplementary Figure S2a). To study the effects of ectoine on the apoptosis of SCC-9 cells, we performed MTT assay. The results showed that ectoine-treated cell proliferation was lower than that of the untreated control cells, although the difference was not statistically significant. However, ectoine had almost no effect on the cell viability (Figure 3c). Moreover, the clonogenic assay showed that the number of colonies formed in ectoine-treated cells was significantly reduced compared with that in untreated cells (Figure 3d and Supplementary Figure S2b). Interestingly, mice injected long-term with HaCaT cells did not induce tumorigenicity of primary skin cells. Henceforth, ectoine reduced the proliferation of skin cell cancer and did not induce tumorigenicity in normal skin cells. 

### 2.4. Ectoine Downregulated Methylation-Related Genes

**Figure 4:**
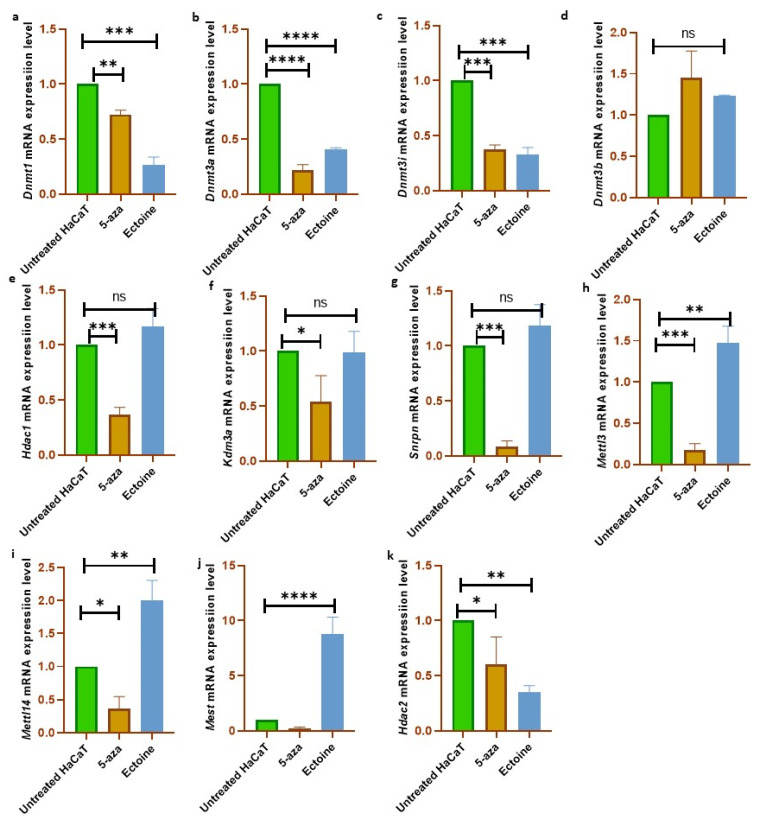
Ectoine modulated the expression level of DNA methylation markers. Bar graphs represent the mRNA expression levels in HaCaT cells treated either with ectoine or 2-aza-deoxycytidine and analyzed using the qPCR technique. The analyzed genes are *Dnmt1* (**a**), *Dnmt3a* (**b**), *Dnmt3b* (**c**), *Dnmt3l* (**d**), *Kdm3a* (**e**), *Mest* (**f**), *Mettl3* (**g**), *Mettl14* (**h**), *Snrpn* (**i**), *Hdac1* (**j**), and *Hdac2* (**k**). (**** *p* < 0.0001), (*** *p* < 0.001), (** *p* < 0.005), (* *p* < 0.01), (ns denotes *p* > 0.05).

To validate the results of the in silico global methylation analysis, the mRNA expression levels of several epigenetic modifications were analyzed (*Dnmt1*, *Dnmt3a*, *Dnmt3b*, *Dnmt3l*, *Hdac1*, *Hdac2*, *Kdm3a*, *Mettl3*, *Mettl14*, *Snrpn*, and *Mest*). We observed that *Dnmt1*, *Dnmt3a*, and *Dnmt3l* were downregulated in cells treated with ectoine for 30 days compared with untreated cells. Similarly, in cells treated with 5-aza, *Dnmt1*, *Dnmt3a*, and *Dnmt3l* were significantly downregulated compared to those in untreated cells (Figure 4a–c). In contrast, *Dnmt3b* mRNA expression levels did not change with ectoine or 5-aza-2-deocycitine treatment (Figure 4d). *Hdac1*, *Kdm3a*, and *Snrpn* mRNA levels were downregulated in 5-aza but not in ectoine-treated cells (Figure 4e–g). Interestingly, *Mettl3*, *Mettl14*, and *Mest* mRNA expression levels were upregulated in ectoine-treated cells, whereas they were downregulated in 5-aza treated cells compared to untreated cells (Figure 4h–j). *Hdac2* mRNA levels were downregulated in ectoine-treated cells compared to 5-aza, and the expression in 5-aza treated cells was lower than in untreated cells (Figure 4k). Thus, ectoine regulates the expression of DNMTs and other epigenetic modification-related mRNA expression levels of other DNA methylation markers.

## 3. Discussion

Epigenetic modifications are primarily driven by DNA methylation and other mechanisms such as acetylation, which is strongly associated with cancer development and ageing. Although several epigenetic drugs have been developed to target cancer, several drawbacks prevent their application owing to serious adverse reactions such as HDACi [1,4]. Environmental stresses and radiations have been linked to DNA hypermethylation. Ectoine protects DNA from environmental stresses and radiation by directly interacting with nearby water molecules [33,38,39]. In the present study, we analyzed global DNA methylation and 5mC levels in extended ectoine-treated skin cells using 5-aza-2-deoxycytidine as a positive control. We also studied the effects of ectoine on skin cell proliferation, tumorigenicity, and the mRNA expression of DNA methylation genes and markers. We observed global ectoine hypomethylation in the skin cells. These results could be explained by another study, which showed that ectoine prevented gene-5-protein (G5P) from bacteriophage Ff from binding to DNA by changing the protein conformation and forming a layer at a distance of 0.5 μm from the DNA [40]. Another recent study showed that ectoine binds to DNA-binding G5P without disturbing the protein structure [41]. This may be because ectoine, through indirect interaction with membrane proteins by preferential exclusion or an unknown mechanism, reduces the production of DNMTs. This was demonstrated by our GO enrichment analysis, which showed that ectoine-treated vs. untreated small molecule binding, nucleotide binding, and ribonucleotide binding-related DEGs were enriched. Our methylome analysis of ectoine-treated cells showed that ectoine significantly reduced methylation levels in almost all regions, which was consistent with the DMP analysis results. Colorectal cancer cells treated with curcumin showed demethylation at specific CpG loci but not global hypomethylation, similar to cells treated with 5-aza-2-deoxycytidine [42]. Our results showed that methylation distribution in the whole genome was reduced compared to that in untreated cells.

In vitro, the results demonstrated that ectoine reduced the percentage of global DNA methylation through reducing the 5-mC percentage and were consistent with the results of in silico analysis of methylome cells, especially at the mCG sequence context in skin cells. A previous study showed that higher 5-mC levels are due to higher DNMT activity in skin cancer cells than in normal cells, which is involved in the silencing of genes that suppress skin tumor development [43]. Furthermore, we observed that ectoine improved cell biological processes such as cell morphogenesis. We also observed that ectoine reduced the proliferation of skin cancer cells but did not induce apoptosis. This is in agreement with a previous study showing that ectoine suppresses the proliferation of head and neck squamous cell carcinoma (HNSCC) [44]. HaCaT cells are sensitive to conversion to tumor cells using different chemicals and under different conditions, such as temperature treatment [45,46,47]. Interestingly, we observed that mice injected with HaCaT cells treated long-term with 140 mM ectoine for 30 weeks did not develop tumors, which demonstrates its safety for skincare applications. Moreover, our results demonstrated that ectoine reduced the expression of Dnmt1, Dnmt3a, and Dnmt3l. This could be due to the interactions of ectoine with membrane proteins or it could be due to a little fraction that entered the cells or an unknown pathway that further needs to be investigated. DNMTs are known to be associated with cancer development, especially hypermethylated tumor suppressor genes and genomic instability [48]. In contrast, our results demonstrated that Hdac1, Kdm3a, and Snrpn mRNA levels were downregulated in 5-aza-2-deoxycytidine but not in ectoine-treated cells. These results demonstrated that ectoine could affect other epigenetic mechanisms such as DNA acetylation and imprinted genes, which require further investigations. Interestingly, our results showed that the mRNA expression levels of Mettl3 and Mettl14 were upregulated in ectoine-treated cells and downregulated in cells treated with 5-aza-2-deoxycytidine. These two enzyme complexes are important for the repair of UV- and X-ray-exposed DNA-damaged sites and subsequent RNA methyl transfer [49]. This agrees with another strategy in which ectoine protects cells against ultraviolet-A (UVA) radiation by inhibiting the proopiomelanocortin (POMC) and tyrosinase pathways in UVA-irradiated HaCaT cells [50]. Moreover, we observed that the mRNA expression levels of the imprinted gene Mest were upregulated in ectoine-treated cells, whereas they were downregulated in 5-aza-2-deoxycytidine. One study showed that 9.6% of patients with oligospermia had Mest methylation; thus, Mest was significantly hypermethylated compared to the control group (3.5%, *p* < 0.001) [51]. From the qPCR analysis results, not all genes were hypomethylated compared to the results of global methylation sequencing findings. This could be due to the lower specificity of global methylation sequencing. Moreover, although these results support that DNA hypomethylation could suppress cancer progress, it is still in contradiction with results that showed that global DNA hypomethylation leads to DNA instability and induces cancer development in human epithelial ovarian cancer [52].

Senescence is a major factor in cancer, nondegenerative diseases, cardiovascular diseases, and several age-related diseases. Methylation marks are inheritable and stable, and there is very little percentage variation over time [53]. Epigenetic modifications, telomere reduction, DNA damage, and mitochondrial function are the primary drivers of aging [54,55]. However, DNA hypermethylation is linked to aging. DNA methylation analysis of human cerebral cortex individuals from different ages showed that age-associated DNA hypermethylation was more prevalent than hypomethylation [56]. Another study showed that the methylator phenotype (CIMP)-associated CGIs or DNA hypermethylation in colon adenocarcinomas provided evidence that age-related CIMP-type hypermethylation promotes carcinogenesis [57]. Importantly, as mentioned in the introduction, ectoine is poorly absorbed by eukaryotic cells, and these activities could be due to ectoine interaction with membrane proteins through preferential exclusion or due to unknown mechanisms. It is worth mentioning that the ectoine dose was relatively large, and the treatment time was relatively long. We cannot rule out the possibility that hypomethylation was a secondary rather than direct effect of the treatment. However, this point needs to be further investigated in future studies. The ability of ectoine to protect DNA, regulate DNA methylation, and suppress tumor development makes it a potential candidate for anti-aging medicine. 

## 4. Materials and Methods

### 4.1. Cell Cultures and Drug Treatments

The human keratinocytes immortal primary cells HaCaT cell line was used in several studies that investigated DNA methylation [58,59]. HaCaT, squamous cell carcinoma (SCC-9), and epidermoid carcinoma (A-431) cells were obtained from the National Center for Cell Sciences (Shanghai, China). These cells were cultivated in Dulbecco’s Modified Eagle’s Medium-F-12 (DMEM-F12) medium enriched with 10% (*v*/*v*) decomplemented fetal bovine serum (FBS) (Invitrogen, Invitrogen, MA, USA) and 100% 100X anti-mycotic/antibiotic solution containing amphotericin B, penicillin, and streptomycin (Gibco, Grand Island, NY, USA). All the samples were incubated under microaerophilic conditions with 100% humidity and 5% CO2 at 37 °C. For the HaCaT cell line, the medium was supplemented with 2 mM L-glutamine and 4.5 g/L glucose, whereas the SSC-9 cell culture was supplemented with hydrocortisone (Sigma, Marlborough, MA, USA) at 400 ng/mL medium and 1% sodium bicarbonate [44]. SCC-9, HaCaT, and A-431 cells were treated with 140 mM (restricted concentration to be used in mouthwash) ectoine [44,50] or 5 μM 5-aza-2-deoxycytidine (Sigma, USA), as modified from a previous study [60]. Regarding 5-aza-2-deoxycytidine, when the cell lines reached 30–40% confluency, they were treated with 5 μM 5-aza-2-deoxycytidine as a positive control and incubated for 72 h in all experiments (cells started to die if treated for longer time). Ectoine was dissolved 0.57 g/mL of water to reach 4 M concentration, and 5-aza-deoxycytidine was dissolved 25 mg/mL in DMSO, sterilized using a 0.22 μm syringe sterile filter, and stored at −20.

### 4.2. Whole-Genome Bisulfite Sequencing and In Silico Methylation Analysis

To investigate the possible effects of ectoine on DNA methylation, HaCaT cells were treated with ectoine for 24 weeks or with 5-aza-2-deoxycytidine for 72 h. The cells were trypsinized and sent to Wuhan Saiwei Biological Technology Co., Ltd. (Wuhan, China) for whole-genome bisulfite sequencing as previously described [61]. Briefly, genomic DNA was extracted using a DNeasy kit (QIAGEN, Hilden, Germany) and randomly fragmented by sonication. The ends were repaired by adding methylated adaptors, isolating the correct fragments, and subjecting them to bisulfite PCR amplification. Finally, WGBS-seq DNA libraries were obtained and subjected to HiSeq. Before data analysis, low-quality sequenced data and linker sequences were removed using the Skewer tool [62], followed by checking the quality control analysis of sequences, which was processed using FastQC [63] for the comparison between sequences. The sequenced data were aligned to the human reference genome using the Bismark tool [64], and sequence comparison was performed using bowtie2 [65]. Only the unique alignments were used for the subsequent analysis. The overall genome methylation and methylation levels of each element of the gene (upstream 2 K, 5-UTR, CDS, intron, 3-UTR, downstream 2 K, and intergenic region) were analyzed to cover the methylated C bases of each gene element. To cover the C bases in the CpG islands, the genome was divided into CpG islands (CGI), Nshelf, Nshore, Sshore, Sshelf, and others, and repeats of CG, CHG, and CHH were calculated. The repeat areas were annotated using ReRepeatMasker 4.1.5. The main categories were SINE, LINE, LTR, and other types of repeats, and the statistics of CG, CHG, and CHH were calculated. The methylated C region was determined from the comparison results of BisMark using a binomial distribution test. DMP CpG sites (where group variations in average (often mean) methylation levels are statistically significant) and differentially methylated regions ((DMR) various biological samples have varied DNA methylation patterns in some genomic locations) were analyzed using the Bioconductor package DSS [66]. For gene ontology (GO) and KEGG, functional enrichment analyses were performed. The sequences were submitted to the NCBI BioSamples (SAMN34441851, SAMN34441852, and SAMN34441853), BioProject (PRJNA970079), and SRA (SUB13191204).

### 4.3. Colony Formation Assay

To study the effects of ectoine on HaCaT colony formation after prolonged treatment, we performed a clonogenic assay as described previously [67]. Cells were seeded at 100 cells/well in a 6-well plate, treated with ectoine, and treated with a culture medium. After 9 days of incubation, the cell colonies were washed with PBS, stained with 0.5% crystal violet in water (Sigma, USA), and washed with ddH2O.

### 4.4. Wound Healing Assay

The treated cell lines (1.6 × 10^6^ cells per well) were seeded in 6-well plates until the cells reached a confluent state, and the cell layer was scratched with a sterile 200 μL pipette tip. The medium and cell debris were aspirated and replaced with 2 mL of fresh DMEM-F12 without antibiotics or FBS. Images of the wounded areas were captured at 0 h and 24 h using a DM13000B light microscope (Leica Microsystems GmbH, Wetzlar, Germany). The wound healing area was calculated as the difference in the area between 0 and 24 h divided by the height of the wound using the ImageJ 1.53t software [68].

### 4.5. MTT Assay

A modified version of a previous study was used to study the effects of ectoine on skin cell viability [28]. SCC-9 and A-431 cells were seeded in 96-well plates at 7500 cells/well, treated with 140 mM ectoine and untreated cells in complete medium, and incubated for 72 h. The medium was removed carefully, and 20 µL of 5 mg/mL MTT was added to each well. Another plate included one set of wells with MTT but no cells (control). The plate was incubated for 3.5 h at 37 °C in a culture hood, the media were removed carefully, and 100 μL DMSO was added. The plate was covered with tinfoil and agitated on an orbital shaker for 30 min, and the absorbance was read at 590 nm with a reference filter of 620 nm using a Tecan microplate reader Infinite^®^ F50 (Tecan, Zürich, Switzerland).

### 4.6. Tumorigenicity In Vivo Experiments

Ectoine has been used in various skincare products. To study the possibility of ectoine-induced tumors in HaCaT keratinocytes, we treated cells with ectoine for 30 weeks and xenografted them into athymic nude mice. This experiment was modified from the previous studies [69,70]. Approximately 5 × 10^6^ HaCaT cells treated with ectoine for 24 weeks were subcutaneously injected into the dorsal flank of athymic nude mice. Mice were divided into an ectoine-treated group, a 5-aza-2-deoxycytidine treated group, and a control group). Ectoine was prepared according to the weight of the mice at 200 mg/kg in ultrapure water and administered every three days. Similarly, deoxycytidine was dissolved in DMSO 2.5 ng/kg and was injected intraperitoneally every three days and continued for 40 days. Mice were euthanized by CO_2_ asphyxiation, as described previously [71,72]. Briefly, the mice were kept in a CO_2_ chamber in small groups and with a medical grade A CO_2_ gas cylinder (3.5 L/min CO_2_ chamber) for 2–3 min until a lack of respiration and faded eyes were observed, followed by 1 min after respiration ceased. The experiments were performed at the Jiangsu University Animal Center, and the experimental procedures were performed in accordance with the principles of the Declaration of Helsinki regulations and guidelines and approved by the Animal Ethics Committee of Jiangsu University.

### 4.7. DNA Methylation Analysis

To validate global DNA methylation, HaCaT cells were treated with either ectoine or 5-aza-2-deoxycytidine and subjected to 5-mC analysis as described previously [73]. Briefly, genomic DNA was extracted from treated HaCaT cells and controls using Axygen^®^ AxyPrep (Axygen, Union City, CA, USA) according to the manufacturer’s instructions. DNA quality was checked using agarose gel, and the concentration was measured using a NanoDrop spectrophotometer (Thermo Scientific, Waltham, MA, USA). An amount of 100 ng from each sample used genomic DNA per reaction to quantify the methylated DNA, which was quantified using a Methylated DNA Quantification Kit Boston Colorimetric, Abcam, ab117128) (Abcam, Cambridge, UK)according to the manufacturer’s instructions. The absorbance was measured using a Tecan microplate reader (Infinite^®^ F50, Tecan, Switzerland) at 450 nm after 10 min. 

### 4.8. Quantitative PCR and Gene Expression Analysis

qRT-PCR analysis was performed as previously described [74]. Briefly, RNA was isolated from HaCaT cells treated with ectoine or 5-aza-2-deoxycytidine using the TRIzol reagent (Invitrogen, USA). Afterward, 3 μg of RNA was converted to cDNA using SuperScript III (Invitrogen, USA) and TA primers, according to the manufacturer’s instructions. For qRT-PCR, 40 ng of the first transcribed DNA strand was amplified using SYBR Fast qPCR Mix (TaKaRa, Shiga, Japan) with primers targeting *Dnmt1*, *Dnmt3a*, *Dnmt3b*, *Dnmt3l*, *Hdac1*, *Hdac2*, *Kdm3a*, *Mettl3*, *Mettl14*, *Snrpn*, and *Mest* genes and *GAPDH* as an internal control [75]. Primer sequences used in this study are listed (Supplementary Table S1).

### 4.9. Statistical Analysis

Statistical analyses were performed using one-way analysis of variance (ANOVA), followed by Tukey’s multiple comparison test. The data are presented as the mean ± standard error (of the mean) from three independent experiments. For data analysis, we used GraphPad Prism 8.0.2 software (Dotmatics, Boston, MA, USA) and ImagJ software [76].

## 5. Conclusions

Ectoine globally demethylates almost all genome elements, including CG, gene, and repeat elements. Moreover, it reduces the 5mC percentage in the genome of skin cells. This is another fascinating mechanism of ectoine DNA protection and demonstrates its therapeutic potential. DNA methylation was mild compared with that in skin cells treated with 5-aza-deoxycytidine, a hypomethylating drug used to treat tumors. Furthermore, in vitro studies of skin cancer cells treated with ectoine have shown mild suppression of skin cancer cell proliferation. Interestingly, long-term treatment of skin cells with ectoine and its use in vivo did not induce tumorigenicity. Moreover, ectoine significantly regulated the expression of DNA methylation genes and markers. Future studies should focus on molecular mechanisms and biophysics to elucidate the manner in which ectoine binds to DNA epigenetic modifiers and how it could regulate other epigenetic mechanisms such as DNA acetylation.

## Data Availability

All data included in this study are available for public use.

## Supplementary Materials

The following supporting information [77,78,79] can be downloaded at: https://www.mdpi.com/article/10.3390/md21120621/s1, Figure S1: Effects of ectoine treatment on skin cell’s pathway and functions.; Figure S2: Ectoine suppressed skin cells proliferation.; Table S1: Primers used in this study.
