# Supplementary material for: Ectoine Globally Hypomethylates DNA in Skin Cells and Suppresses Cancer Proliferation
Source: Mar Drugs. 2023 Nov 29;21(12):621. doi: 10.3390/md21120621 (PMC10744768; doi:10.3390/md21120621)
Supplement: Supplementary file 1 [file marinedrugs-21-00621-s001.zip › marinedrugs-2644685-supplementary.pdf]

GO and KEGG functional enrichment analysis.

For gene ontology (GO) function analysis (includes biological processes (BP) molecular functions (MF) cellular components (CC)) topGO software [78] was used to annotate and classify the functions. The functional enrichment for the target gene was listed and used Fisher's exact test to calculate the representative GO function set in the target gene list. The

P value of whether the gene list is significantly enriched, and then the P value is corrected by Benjamini & Hochberg's multiple tests to get the false discovery rate (FDR) [79]. The KEGG pathway functional enrichment analysis [80] from the differentially expressed genes (DEGs) was similar to the GO functional enrichment analysis, and the significant enrichment pathways are screened according to *p value* ≤ 0.05.

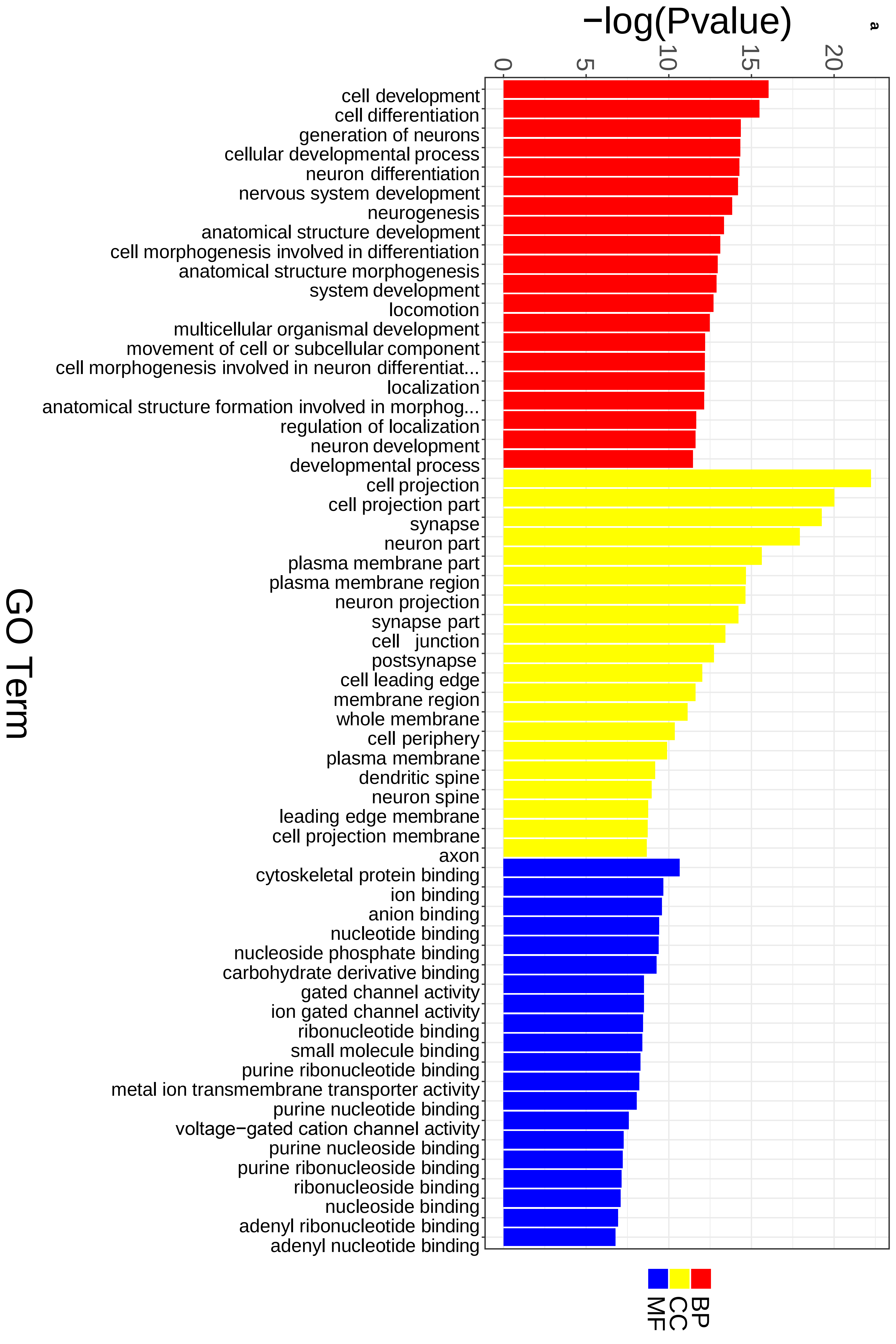
a
−log(Pvalue)
0
5
10
15
20
GO Term
cell development
cell differentiation
generation of neurons
cellular developmental process
neuron differentiation
nervous system development
neurogenesis
anatomical structure development
cell morphogenesis involved in differentiation
anatomical structure morphogenesis
system development
locomotion
multicellular organismal development
movement of cell or subcellular component
cell morphogenesis involved in neuron differentiat...
localization
anatomical structure formation involved in morphog...
regulation of localization
neuron development
developmental process
cell projection
cell projection part
synapse
neuron part
plasma membrane part
plasma membrane region
neuron projection
synapse part
cell junction
postsynapse
cell leading edge
membrane region
whole membrane
cell periphery
plasma membrane
dendritic spine
neuron spine
leading edge membrane
cell projection membrane
axon
cytoskeletal protein binding
ion binding
anion binding
nucleotide binding
nucleoside phosphate binding
carbohydrate derivative binding
gated channel activity
ion gated channel activity
ribonucleotide binding
small molecule binding
purine ribonucleotide binding
metal ion transmembrane transporter activity
purine nucleotide binding
voltage−gated cation channel activity
purine nucleoside binding
purine ribonucleoside binding
ribonucleoside binding
nucleoside binding
adenyl ribonucleotide binding
adenyl nucleotide binding
BP
CC
MF

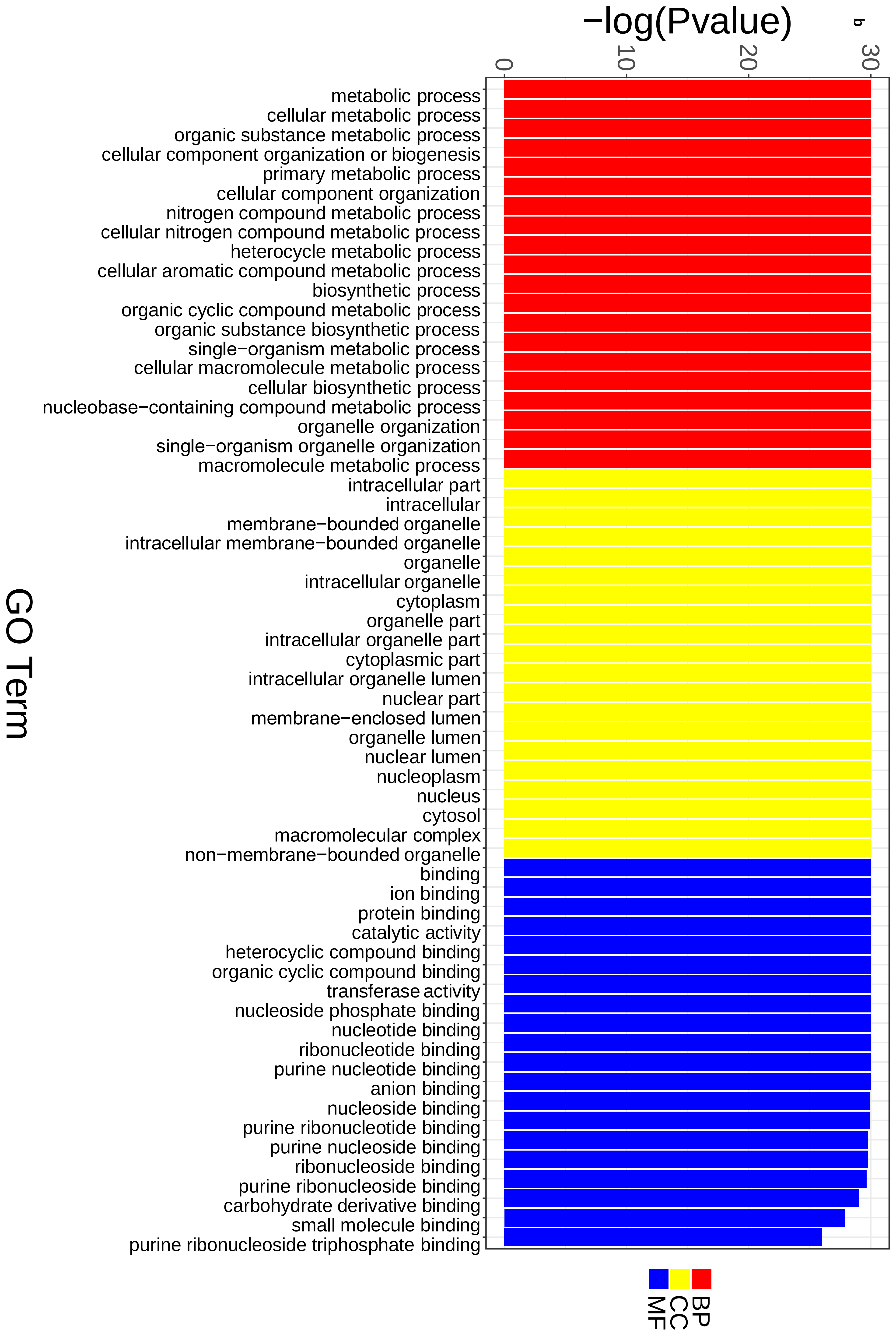
b
−log(Pvalue)
0
10
20
30
GO Term
metabolic process
cellular metabolic process
organic substance metabolic process
cellular component organization or biogenesis
primary metabolic process
cellular component organization
nitrogen compound metabolic process
cellular nitrogen compound metabolic process
heterocycle metabolic process
cellular aromatic compound metabolic process
biosynthetic process
organic cyclic compound metabolic process
organic substance biosynthetic process
single−organism metabolic process
cellular macromolecule metabolic process
cellular biosynthetic process
nucleobase−containing compound metabolic process
organelle organization
single−organism organelle organization
macromolecule metabolic process
intracellular part
intracellular
membrane−bounded organelle
intracellular membrane−bounded organelle
organelle
intracellular organelle
cytoplasm
organelle part
intracellular organelle part
cytoplasmic part
intracellular organelle lumen
nuclear part
membrane−enclosed lumen
organelle lumen
nuclear lumen
nucleoplasm
nucleus
cytosol
macromolecular complex
non−membrane−bounded organelle
binding
ion binding
protein binding
catalytic activity
heterocyclic compound binding
organic cyclic compound binding
transferase activity
nucleoside phosphate binding
nucleotide binding
ribonucleotide binding
purine nucleotide binding
anion binding
nucleoside binding
purine ribonucleotide binding
purine nucleoside binding
ribonucleoside binding
purine ribonucleoside binding
carbohydrate derivative binding
small molecule binding
purine ribonucleoside triphosphate binding
BP
CC
MF

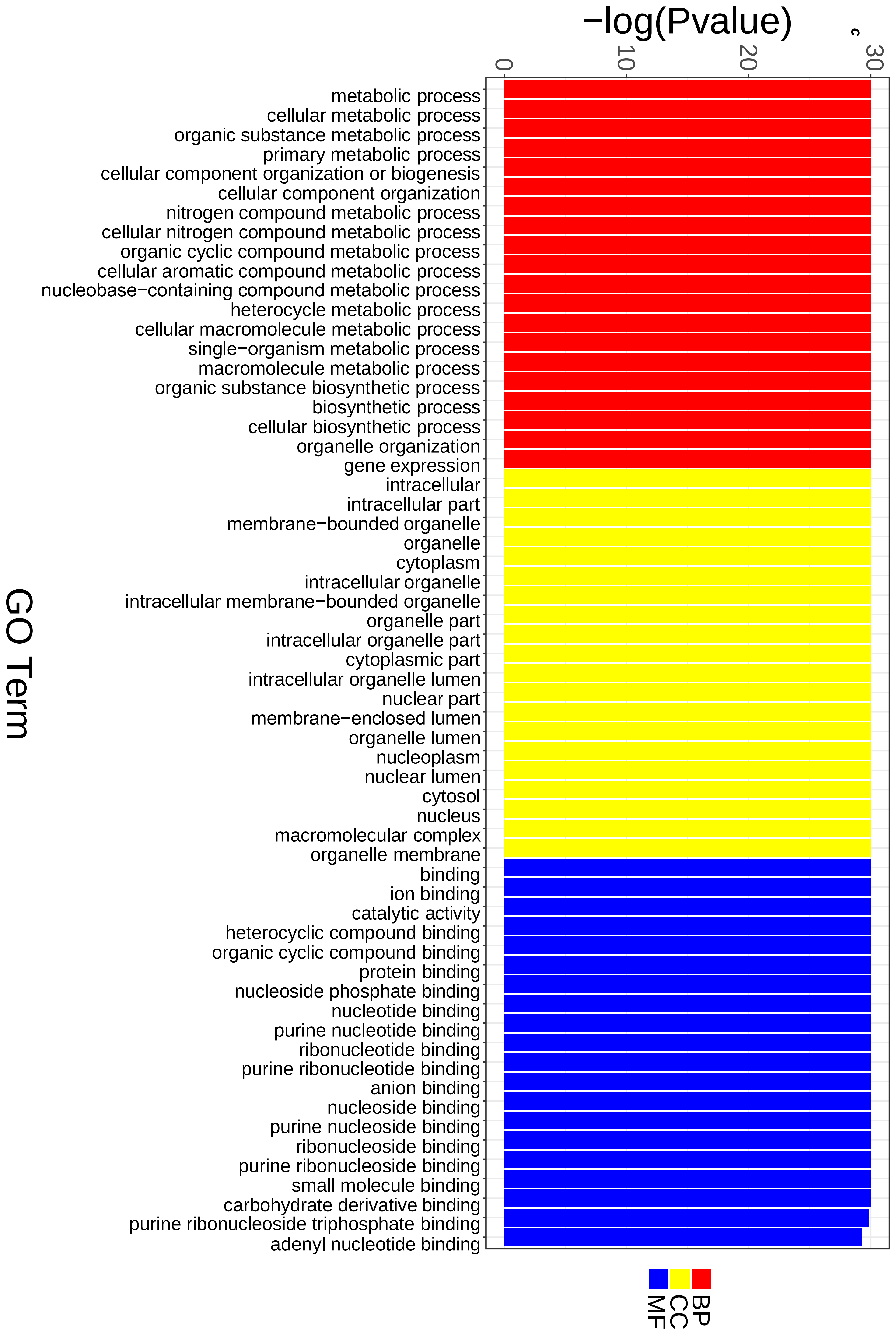
−log(Pvalue)
c
0
10
20
30
GO Term
metabolic process
cellular metabolic process
organic substance metabolic process
primary metabolic process
cellular component organization or biogenesis
cellular component organization
nitrogen compound metabolic process
cellular nitrogen compound metabolic process
organic cyclic compound metabolic process
cellular aromatic compound metabolic process
nucleobase−containing compound metabolic process
heterocycle metabolic process
cellular macromolecule metabolic process
single−organism metabolic process
macromolecule metabolic process
organic substance biosynthetic process
biosynthetic process
cellular biosynthetic process
organelle organization
gene expression
intracellular
intracellular part
membrane−bounded organelle
organelle
cytoplasm
intracellular organelle
intracellular membrane−bounded organelle
organelle part
intracellular organelle part
cytoplasmic part
intracellular organelle lumen
nuclear part
membrane−enclosed lumen
organelle lumen
nucleoplasm
nuclear lumen
cytosol
nucleus
macromolecular complex
organelle membrane
binding
ion binding
catalytic activity
heterocyclic compound binding
organic cyclic compound binding
protein binding
nucleoside phosphate binding
nucleotide binding
purine nucleotide binding
ribonucleotide binding
purine ribonucleotide binding
anion binding
nucleoside binding
purine nucleoside binding
ribonucleoside binding
purine ribonucleoside binding
small molecule binding
carbohydrate derivative binding
purine ribonucleoside triphosphate binding
adenyl nucleotide binding
BP
CC
MF

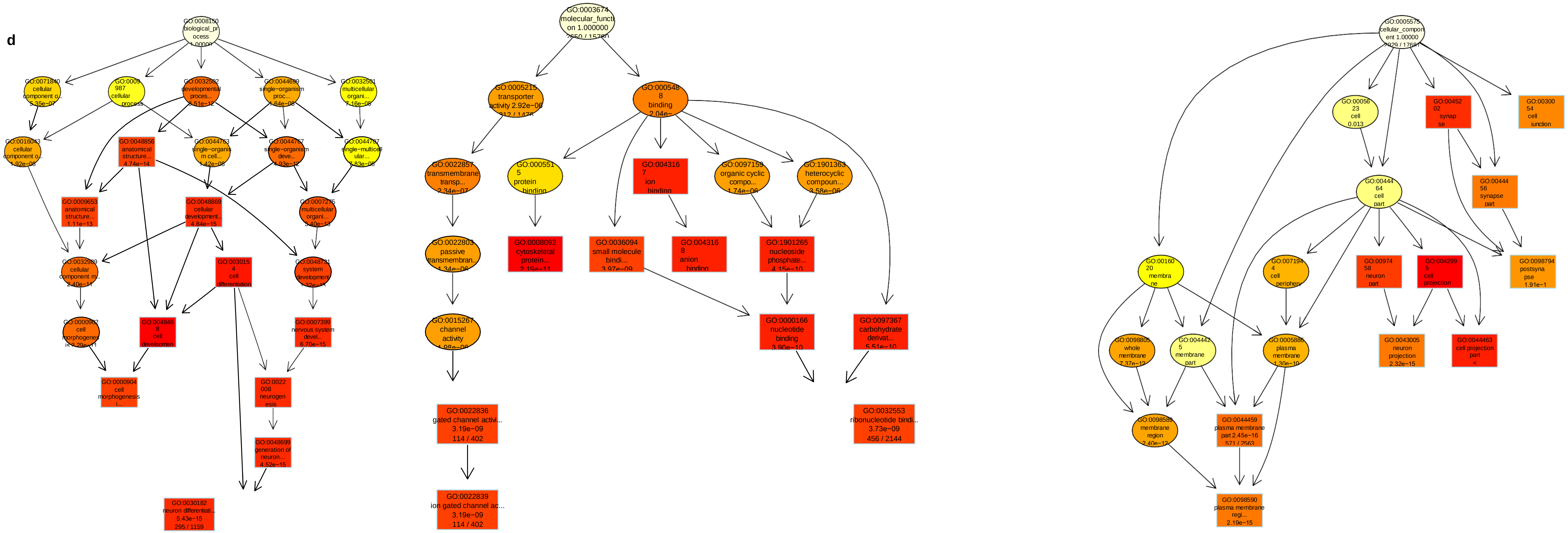
d
GO:0008150 biological_process
GO:0071840 cellular component o... 5.35e−07
GO:0009987 cellular process
GO:0032502 developmental proces... 8.51e−12
GO:0044699 single−organism proc... 1.64e−08
GO:0032501 multicellular organi... 7.16e−05
GO:0016043 cellular component o... 1.92e−08
GO:0048856 anatomical structure... 4.74e−14
GO:0044763 single−organism cell... 1.42e−08
GO:0044767 single−organism deve... 4.93e−12
GO:0044707 single−multicellular... 7.83e−05
GO:0009653 anatomical structure... 1.11e−13
GO:0048869 cellular development... 4.84e−15
GO:0007275 multicellular organi... 3.40e−13
GO:0032989 cellular component m... 2.40e−11
GO:0030154 cell differentiation
GO:0048731 system development
GO:0000902 cell morphogenesis
GO:0048468 cell developmen
GO:0007399 nervous system devel... 6.70e−15
GO:0000904 cell morphogenesis i...
GO:0022008 neurogenesis
GO:0048699 generation of neuron... 4.52e−15
GO:0030182 neuron differentiati... 5.43e−15 295 / 1159
GO:0003674 molecular_function 1.000000
GO:0005215 transporter activity 2.92e−06
GO:0005488 binding
GO:0022857 transmembrane transp... 2.34e−07
GO:0005515 protein binding
GO:0043167 ion binding
GO:0097159 organic cyclic compo... 1.74e−06
GO:1901363 heterocyclic compoun... 3.58e−06
GO:0022803 passive transmembran... 1.34e−06
GO:0008092 cytoskeletal protein... 2.19e−11
GO:0036094 small molecule bindi... 3.97e−09
GO:0043168 anion binding
GO:1901265 nucleoside phosphate... 4.15e−10
GO:0015267 channel activity
GO:0000166 nucleotide binding 3.90e−10
GO:0097367 carbohydrate derivat... 5.51e−10
GO:0022836 gated channel activi... 3.19e−09 114 / 402
GO:0032553 ribonucleotide bindi... 3.73e−09 456 / 2144
GO:0022839 ion gated channel ac... 3.19e−09 114 / 402
GO:0005575 cellular_component 1.00000
GO:0005623 cell 0.013
GO:0045202 synapse
GO:0030054 cell junction
GO:0044464 cell part
GO:0044456 synapse part
GO:0016020 membrane
GO:0071944 cell periphery
GO:0097458 neuron part
GO:0042995 cell projection
GO:0098794 postsynapse 1.91e−1
GO:0098805 whole membrane 7.37e−12
GO:0044425 membrane part
GO:0005886 plasma membrane 1.30e−10
GO:0043005 neuron projection 2.32e−15
GO:0044463 cell projection part
GO:0098589 membrane region 2.40e−12
GO:0044459 plasma membrane part 2.45e−16 571 / 2563
GO:0098590 plasma membrane regi... 2.19e−15

e

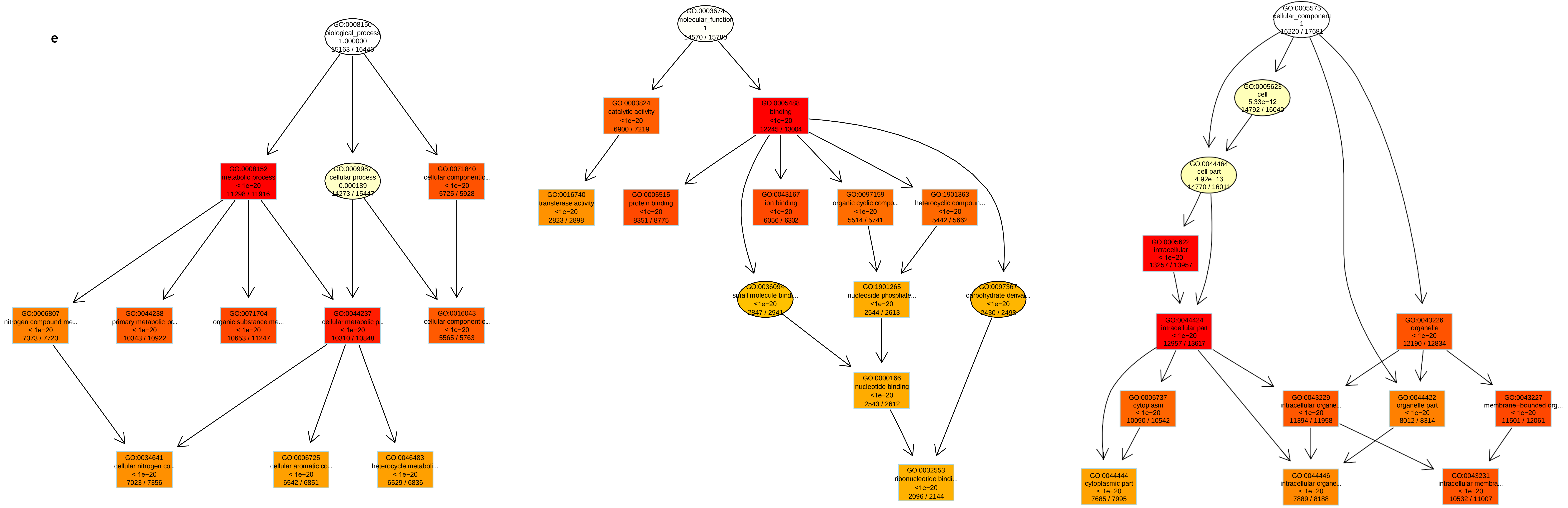

GO:0008150
biological_process
1.000000
15163 / 16446
GO:0008152
metabolic process
< 1e−20
11298 / 11916
GO:0009987
cellular process
0.000189
14273 / 15447
GO:0071840
cellular component o...
< 1e−20
5725 / 5928
GO:0006807
nitrogen compound me...
< 1e−20
7373 / 7723
GO:0044238
primary metabolic pr...
< 1e−20
10343 / 10922
GO:0071704
organic substance me...
< 1e−20
10653 / 11247
GO:0044237
cellular metabolic p...
< 1e−20
10310 / 10848
GO:0016043
cellular component o...
< 1e−20
5565 / 5763
GO:0034641
cellular nitrogen co...
< 1e−20
7023 / 7356
GO:0006725
cellular aromatic co...
< 1e−20
6542 / 6851
GO:0046483
heterocycle metaboli...
< 1e−20
6529 / 6836
GO:0003674
molecular_function
1
14570 / 15780
GO:0003824
catalytic activity
<1e−20
6900 / 7219
GO:0005488
binding
<1e−20
12245 / 13004
GO:0016740
transferase activity
<1e−20
2823 / 2898
GO:0005515
protein binding
<1e−20
8351 / 8775
GO:0043167
ion binding
<1e−20
6056 / 6302
GO:0097159
organic cyclic compo...
<1e−20
5514 / 5741
GO:1901363
heterocyclic compoun...
<1e−20
5442 / 5662
GO:0036094
small molecule bindi...
<1e−20
2847 / 2941
GO:1901265
nucleoside phosphate...
<1e−20
2544 / 2613
GO:0097367
carbohydrate derivat...
<1e−20
2430 / 2498
GO:0000166
nucleotide binding
<1e−20
2543 / 2612
GO:0032553
ribonucleotide bindi...
<1e−20
2096 / 2144
GO:0005575
cellular_component
1
16220 / 17681
GO:0005623
cell
5.33e−12
14792 / 16040
GO:0044464
cell part
4.92e−13
14770 / 16011
GO:0005622
intracellular
< 1e−20
13257 / 13957
GO:0044424
intracellular part
< 1e−20
12957 / 13617
GO:0043226
organelle
< 1e−20
12190 / 12834
GO:0005737
cytoplasm
< 1e−20
10090 / 10542
GO:0043229
intracellular organe...
< 1e−20
11394 / 11958
GO:0044422
organelle part
< 1e−20
8012 / 8314
GO:0043227
membrane−bounded org...
< 1e−20
11501 / 12061
GO:0044444
cytoplasmic part
< 1e−20
7685 / 7995
GO:0044446
intracellular organe...
< 1e−20
7889 / 8188
GO:0043231
intracellular membra...
< 1e−20
10532 / 11007

g

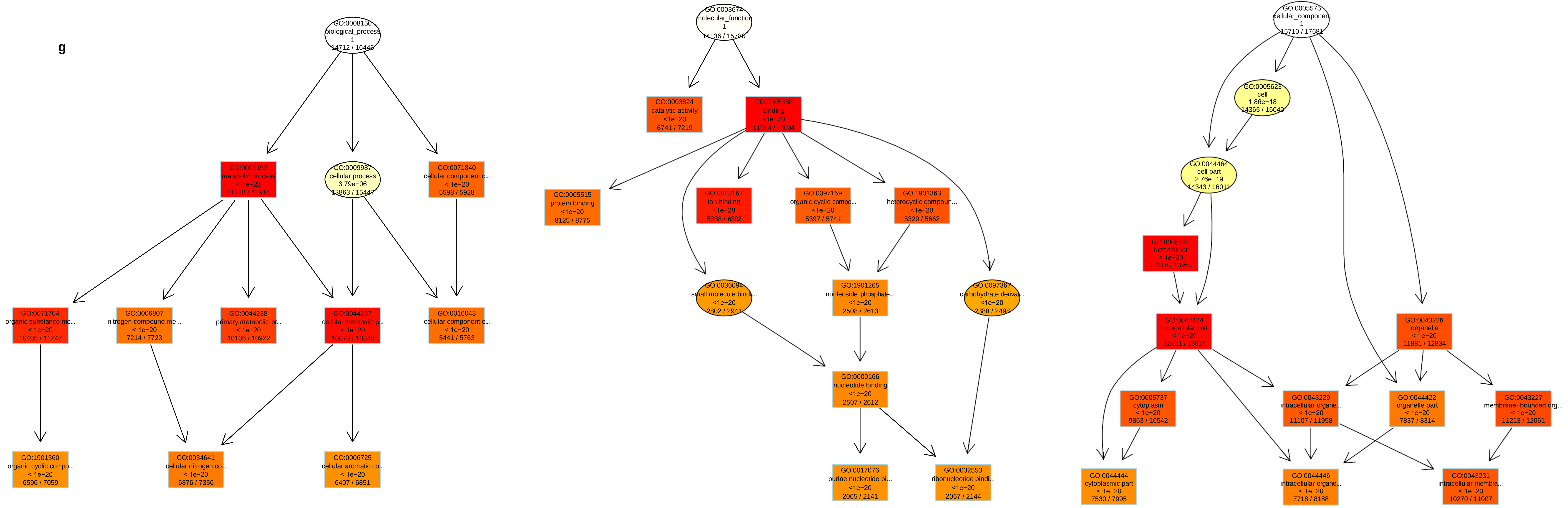

GO:0008150 biological_process 1 14712 / 16446
GO:0008152 metabolic process < 1e−20 11018 / 11916
GO:0009987 cellular process 3.79e−06 13863 / 15447
GO:0071840 cellular component o... < 1e−20 5598 / 5928
GO:0071704 organic substance me... < 1e−20 10405 / 11247
GO:0006807 nitrogen compound me... < 1e−20 7214 / 7723
GO:0044238 primary metabolic pr... < 1e−20 10106 / 10922
GO:0044237 cellular metabolic p... < 1e−20 10070 / 10848
GO:0016043 cellular component o... < 1e−20 5441 / 5763
GO:1901360 organic cyclic compo... < 1e−20 6596 / 7059
GO:0034641 cellular nitrogen co... < 1e−20 6876 / 7356
GO:0006725 cellular aromatic co... < 1e−20 6407 / 6851
GO:0003674 molecular_function 1 14136 / 15780
GO:0003824 catalytic activity <1e−20 6741 / 7219
GO:0005488 binding <1e−20 11914 / 13004
GO:0005515 protein binding <1e−20 8125 / 8775
GO:0043167 ion binding <1e−20 5938 / 6302
GO:0097159 organic cyclic compo... <1e−20 5397 / 5741
GO:1901363 heterocyclic compoun... <1e−20 5329 / 5662
GO:0036094 small molecule bindi... <1e−20 2802 / 2941
GO:1901265 nucleoside phosphate... <1e−20 2508 / 2613
GO:0097367 carbohydrate derivat... <1e−20 2388 / 2498
GO:0000166 nucleotide binding <1e−20 2507 / 2612
GO:0017076 purine nucleotide bi... <1e−20 2065 / 2141
GO:0032553 ribonucleotide bindi... <1e−20 2067 / 2144
GO:0005575 cellular_component 1 15710 / 17681
GO:0005623 cell 1.86e−18 14365 / 16040
GO:0044464 cell part 2.76e−19 14343 / 16011
GO:0005622 intracellular < 1e−20 12915 / 13957
GO:0044424 intracellular part < 1e−20 12621 / 13617
GO:0043226 organelle < 1e−20 11881 / 12834
GO:0005737 cytoplasm < 1e−20 9863 / 10542
GO:0043229 intracellular organe... < 1e−20 11107 / 11958
GO:0044422 organelle part < 1e−20 7837 / 8314
GO:0043227 membrane−bounded org... < 1e−20 11213 / 12061
GO:0044444 cytoplasmic part < 1e−20 7530 / 7995
GO:0044446 intracellular organe... < 1e−20 7718 / 8188
GO:0043231 intracellular membra... < 1e−20 10270 / 11007

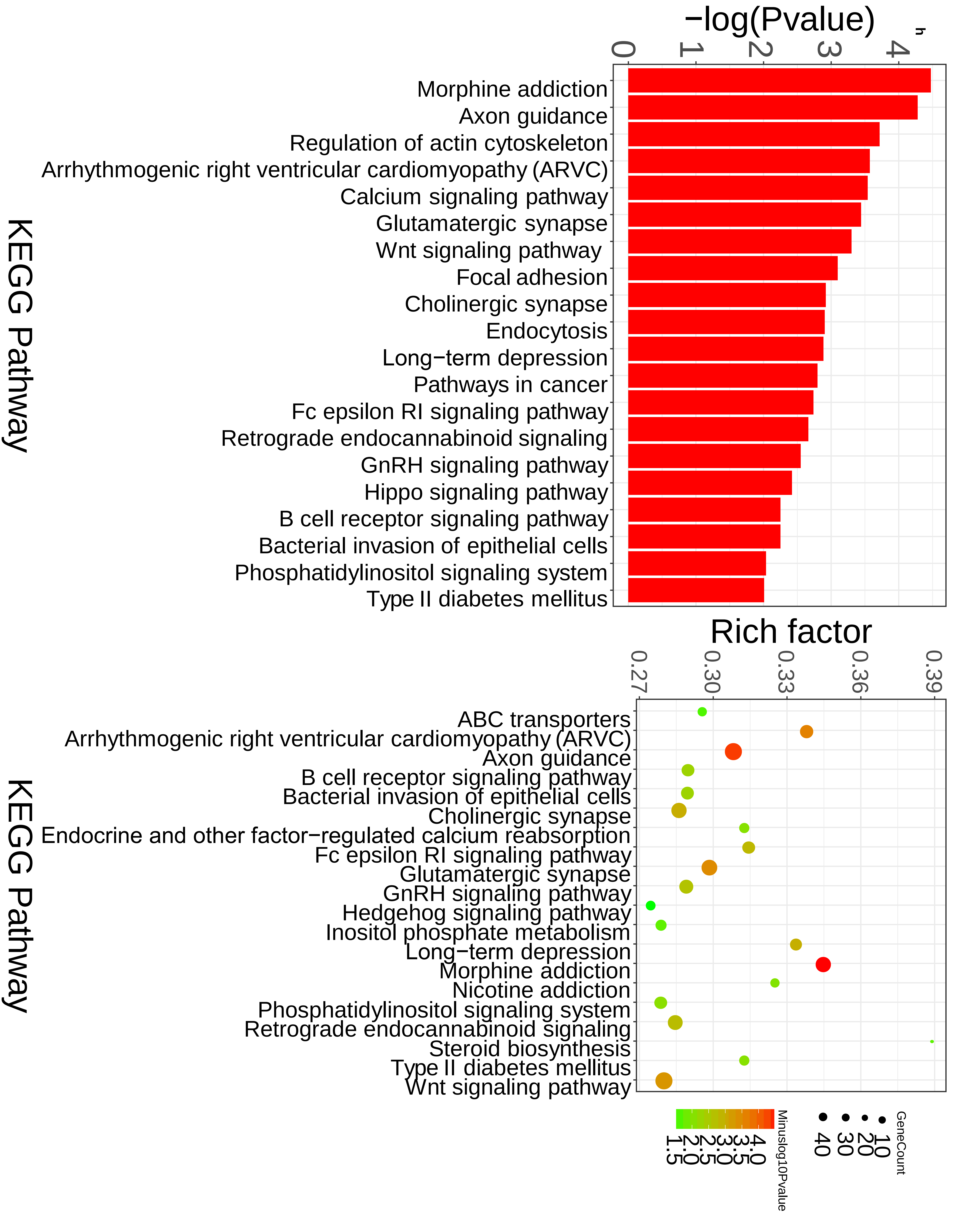
−log(Pvalue)
0
1
2
3
4
KEGG Pathway
Morphine addiction
Axon guidance
Regulation of actin cytoskeleton
Arrhythmogenic right ventricular cardiomyopathy (ARVC)
Calcium signaling pathway
Glutamatergic synapse
Wnt signaling pathway
Focal adhesion
Cholinergic synapse
Endocytosis
Long−term depression
Pathways in cancer
Fc epsilon RI signaling pathway
Retrograde endocannabinoid signaling
GnRH signaling pathway
Hippo signaling pathway
B cell receptor signaling pathway
Bacterial invasion of epithelial cells
Phosphatidylinositol signaling system
Type II diabetes mellitus
Rich factor
0.27
0.30
0.33
0.36
0.39
KEGG Pathway
ABC transporters
Arrhythmogenic right ventricular cardiomyopathy (ARVC)
Axon guidance
B cell receptor signaling pathway
Bacterial invasion of epithelial cells
Cholinergic synapse
Endocrine and other factor−regulated calcium reabsorption
Fc epsilon RI signaling pathway
Glutamatergic synapse
GnRH signaling pathway
Hedgehog signaling pathway
Inositol phosphate metabolism
Long−term depression
Morphine addiction
Nicotine addiction
Phosphatidylinositol signaling system
Retrograde endocannabinoid signaling
Steroid biosynthesis
Type II diabetes mellitus
Wnt signaling pathway
Minuslog10Pvalue
1.5
2.0
2.5
3.0
3.5
4.0
GeneCount
10
20
30
40

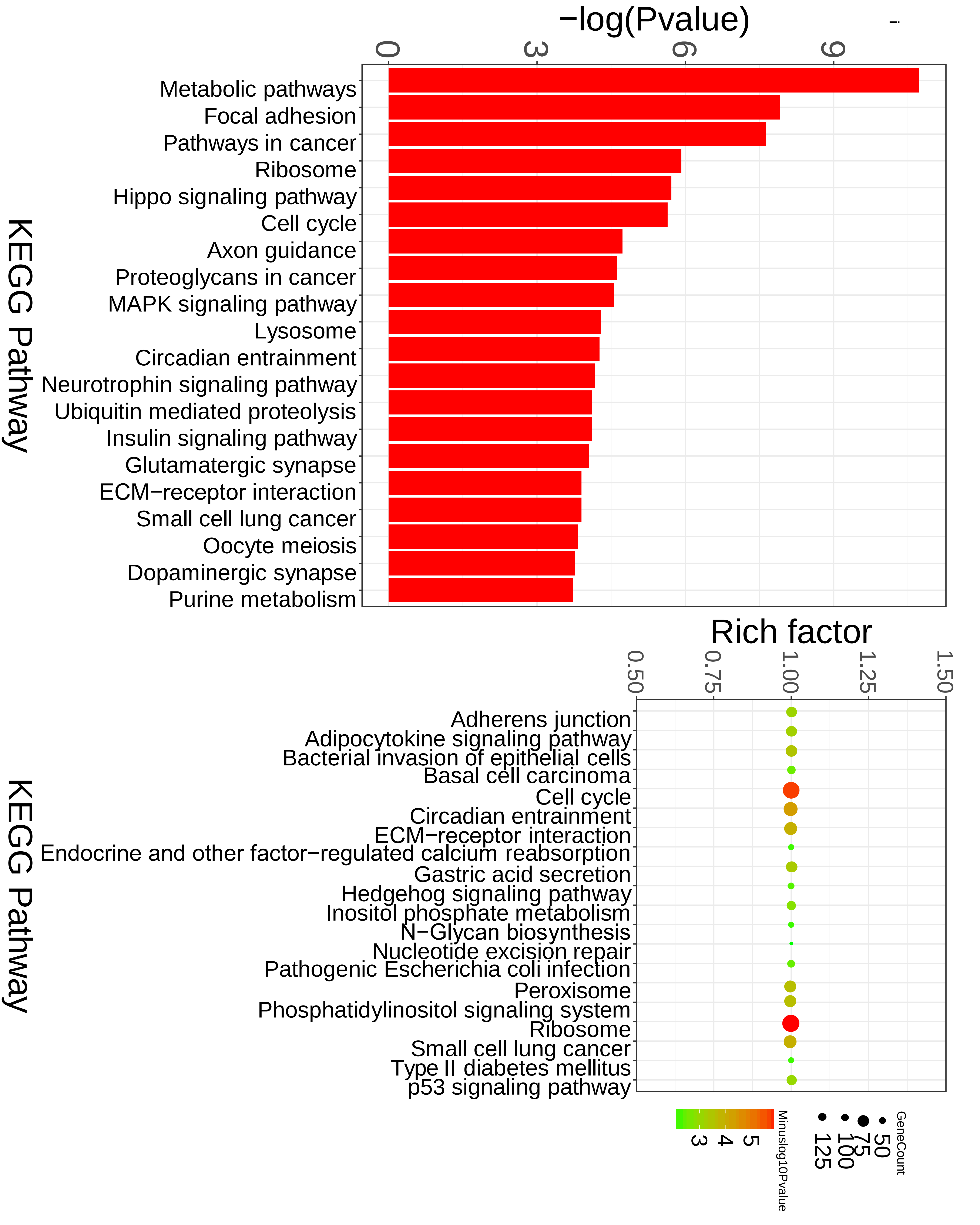
−log(Pvalue)
0
3
6
9
KEGG Pathway
Metabolic pathways
Focal adhesion
Pathways in cancer
Ribosome
Hippo signaling pathway
Cell cycle
Axon guidance
Proteoglycans in cancer
MAPK signaling pathway
Lysosome
Circadian entrainment
Neurotrophin signaling pathway
Ubiquitin mediated proteolysis
Insulin signaling pathway
Glutamatergic synapse
ECM−receptor interaction
Small cell lung cancer
Oocyte meiosis
Dopaminergic synapse
Purine metabolism
Rich factor
0.50
0.75
1.00
1.25
1.50
KEGG Pathway
Adherens junction
Adipocytokine signaling pathway
Bacterial invasion of epithelial cells
Basal cell carcinoma
Cell cycle
Circadian entrainment
ECM−receptor interaction
Endocrine and other factor−regulated calcium reabsorption
Gastric acid secretion
Hedgehog signaling pathway
Inositol phosphate metabolism
N−Glycan biosynthesis
Nucleotide excision repair
Pathogenic Escherichia coli infection
Peroxisome
Phosphatidylinositol signaling system
Ribosome
Small cell lung cancer
Type II diabetes mellitus
p53 signaling pathway
Minuslog10Pvalue
3
4
5
GeneCount
50
75
100
125

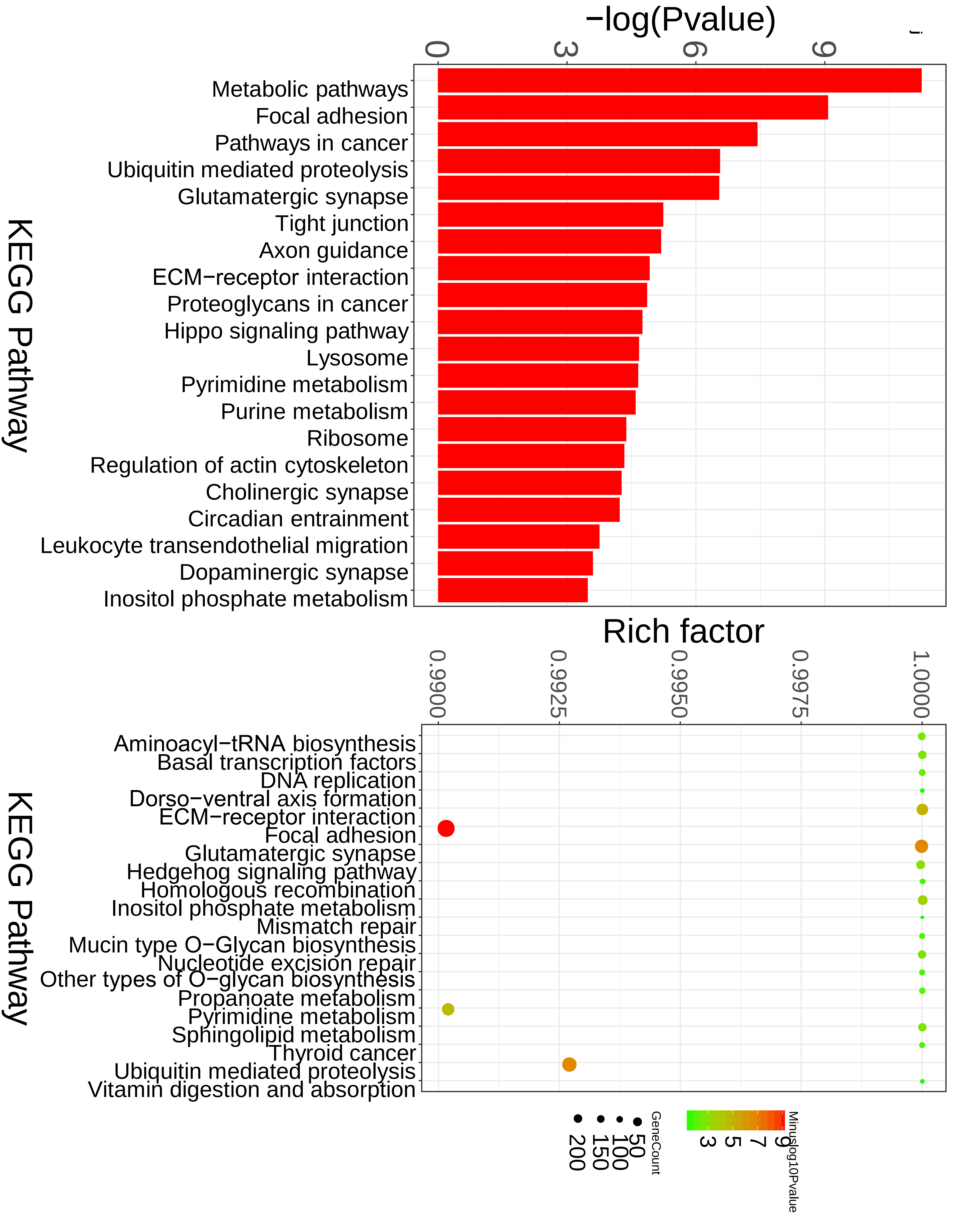
−log(Pvalue)
0
3
6
9
KEGG Pathway
Metabolic pathways
Focal adhesion
Pathways in cancer
Ubiquitin mediated proteolysis
Glutamatergic synapse
Tight junction
Axon guidance
ECM−receptor interaction
Proteoglycans in cancer
Hippo signaling pathway
Lysosome
Pyrimidine metabolism
Purine metabolism
Ribosome
Regulation of actin cytoskeleton
Cholinergic synapse
Circadian entrainment
Leukocyte transendothelial migration
Dopaminergic synapse
Inositol phosphate metabolism
Rich factor
0.9900
0.9925
0.9950
0.9975
1.0000
KEGG Pathway
Aminoacyl−tRNA biosynthesis
Basal transcription factors
DNA replication
Dorso−ventral axis formation
ECM−receptor interaction
Focal adhesion
Glutamatergic synapse
Hedgehog signaling pathway
Homologous recombination
Inositol phosphate metabolism
Mismatch repair
Mucin type O−Glycan biosynthesis
Nucleotide excision repair
Other types of O−glycan biosynthesis
Propanoate metabolism
Pyrimidine metabolism
Sphingolipid metabolism
Thyroid cancer
Ubiquitin mediated proteolysis
Vitamin digestion and absorption
GeneCount
50
100
150
200
Minuslog10Pvalue
3
5
7
9

Supplementary figure S1: ***Effects of ectoine treatment on skin cell's pathway and functions***.

Histogram represents the GO enrichment functional analysis to the difference between functional pathways between ectoine/or 5-aza-2-deoxycytidine treated cells and untreated cells. The ectoine vs untreated showed lower expression in the Biological Processes (BP) Molecular Functions (MF) Cellular Components (CC) (a) compared to 5-aza vs untreated (b) and 5-aza vs ectoine (c). Note: The ordinate represents -log10 (*p value*), and the abscissa represents the names of significantly enriched GOs (up to the top 10 GOs for each category). Similarly, using treemap ectoine vs untreated (d) the ion binding, small molecule binding, nucleotide binding and ribonucleotide binding related DEGs were significantly increased compared to 5-aza vs untreated (e) and 5-aza vs ectoine (f). Moreover, ectoine improved cell's biological processes such as cell morphogenesis,

Note: There are three subtrees in total, Biological_process subtree, molecular_function subtree, and cellular_component subtree, the highlighted box represents significant enrichment GO.

KEEG functional analysis showed that ectoine vs untreated (g) reduced the overall expression of genes compared to 5-aza vs untreated (h) and 5-aza vs ectoine (i). ectoine induced the expression of morphine addiction.

Note: The abscissa represents the name of the significantly enriched KEGG pathway, and the ordinate represents -log10(*p value*). Red bars indicate significant pathways and blue bars indicate insignificant pathways. Display up to the top 20 KEGG pathways. The ordinate represents the Rich factor (the ratio of the total number of genes belonging to this KEGG pathway to the total number of genes belonging to this KEGG pathway). The larger the Rich factor, the greater the degree of enrichment, and the abscissa represents -log10 (*p value*). According to the sorting information of the Rich factor, at most, the top 20 KEGG pathways are displayed.

a

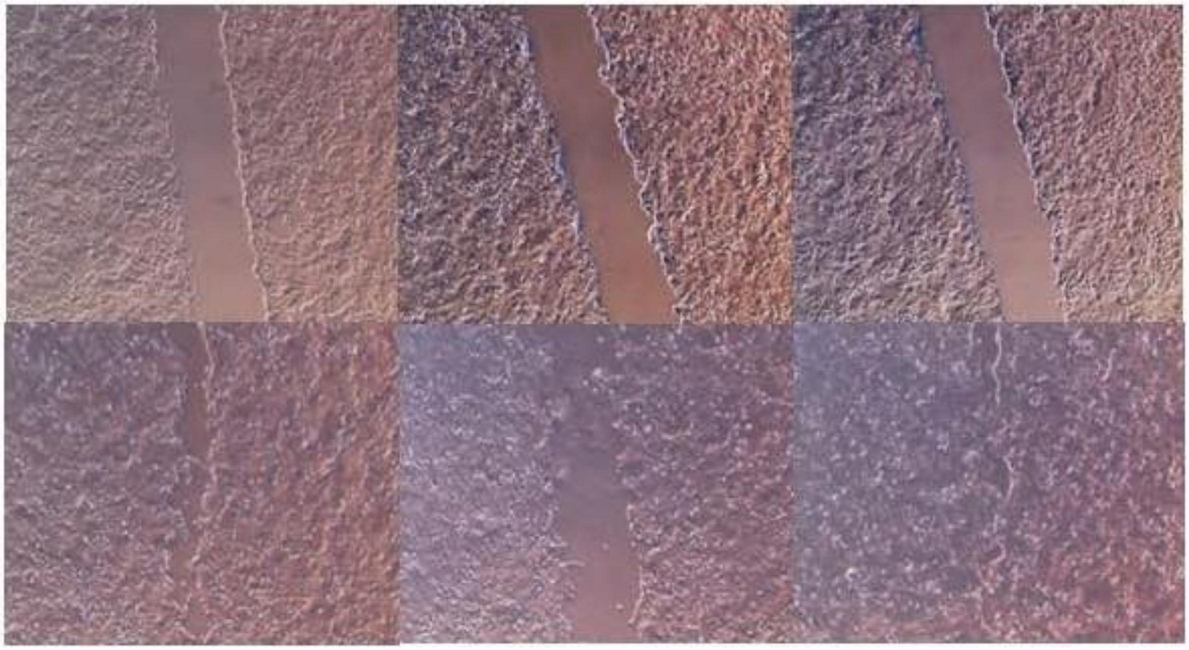

b

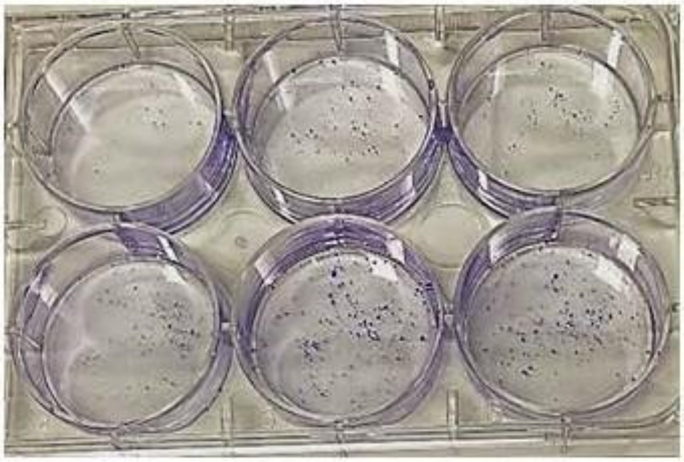

Suppl. figure S2: ***Ectoine suppressed skin cells proliferation.*** Wound healing assay in A-431 cell lines treated with ectoine or 5-aza (A) and colonogenic assay for HaCaT cell line treated with ectoine (B).
